# Circulating Endothelial-Associated Biomarker Responses During Beating-Heart Partial Versus Cardioplegic Total Cardiopulmonary Bypass in Juvenile Pigs: An Exploratory Comparative Study

**DOI:** 10.3390/vetsci13090861

**Published:** 2026-08-25

**Authors:** Hiromu Udagawa, Yuji Hamamoto, Eunryel Nam, Minoru Okamoto, Masafumi Komatsu, Mitsuhiro Isaka

**Affiliations:** 1 Laboratory of Companion Animal Surgery, Department of Small Animal Clinical Sciences, School of Veterinary Medicine, Rakuno Gakuen University, 582 Bunkyodai Midorimachi, Ebetsu 069-8501, Hokkaido, Japan; s22341001@stu.rakuno.ac.jp (H.U.);; 2 Laboratory of Clinical Pathology, Department of Small Animal Clinical Sciences, School of Veterinary Medicine, Rakuno Gakuen University, 582 Bunkyodai Midorimachi, Ebetsu 069-8501, Hokkaido, Japan; mokamoto@rakuno.ac.jp

**Keywords:** endothelial activation, vascular cell adhesion molecule-1, pulsatility, shear stress, porcine model

## Abstract

Cardiopulmonary bypass (CPB) alters blood flow patterns and may induce endothelial activation; however, the effects of different CPB configurations on endothelial-associated biomarkers remain unclear. In this exploratory pilot study, we compared circulating biomarkers and tissue immunohistochemical findings between beating-heart partial CPB and cardioplegic total CPB in juvenile pigs. Ten 8-week-old female crossbred piglets underwent partial CPB with continued cardiac beating (*n* = 5) or total CPB with aortic cross-clamping and cardioplegic arrest (*n* = 5) for 60 min. Serum vascular cell adhesion molecule-1 (VCAM-1), a disintegrin and metalloproteinase-17 (ADAM-17), and thrombospondin-1 (TSP-1) concentrations were measured using an enzyme-linked immunosorbent assay, and tissue VCAM-1 and platelet endothelial cell adhesion molecule-1 (PECAM-1) were assessed immunohistochemically. At the end of the CPB procedure, serum VCAM-1 was higher in the total CPB group than in the partial CPB group (581 [339–759] ng/mL vs. 239 [224–304] ng/mL; |rank-biserial r| = 0.84 (95%CI, 0.36 to 1.00); Hodges–Lehmann estimate of the location shift = 341.7 ng/mL, 95% CI, 5.1–607.0 ng/mL; *p* = 0.03), whereas ADAM-17 and TSP-1 and tissue findings showed no consistent intergroup differences. Total CPB was associated with an early increase in circulating VCAM-1. Because the CPB configurations differed in several physiological and procedural factors, this response should be interpreted as configuration-associated rather than as evidence of an isolated effect of reduced pulsatility.

## Simple Summary

Cardiopulmonary bypass (CPB) is essential for many cardiac surgical procedures but may promote inflammation and postoperative organ dysfunction. Blood flow patterns during CPB depend on whether the heart continues beating or is temporarily arrested. Because vascular endothelial cells respond to changes in flow and shear stress, these differences may influence early endothelial-associated responses. In this exploratory study, juvenile pigs underwent either beating-heart partial CPB or cardioplegic total CPB for 60 min. At the end of CPB, the circulating concentration of vascular cell adhesion molecule-1 was higher in animals undergoing total CPB, whereas the other circulating biomarkers and tissue immunostaining findings did not differ consistently between groups. The two CPB configurations differed not only in native pulsatility but also in aortic cross-clamping, cardioplegia, myocardial ischemia–reperfusion, and native cardiac output. Therefore, the observed response cannot be attributed to reduced pulsatility alone. These preliminary findings suggest that CPB configuration may influence an early circulating endothelial-associated biomarker response and support further studies using larger cohorts and direct measurements of pulsatility and wall shear stress.

## 1. Introduction

Vascular endothelial cells continuously sense the mechanical forces generated by flowing blood. Among these forces, wall shear stress is determined by blood viscosity and the velocity gradient adjacent to the endothelial surface, whereas its biological effects also depend on temporal pattern, directionality, and pulsatility [1,2,3,4,5]. Under physiological conditions, pulsatile flow contributes to vascular homeostasis by regulating endothelial barrier function, inflammatory signaling, leukocyte adhesion, and thromboregulatory pathways. Conversely, low oscillatory or otherwise disturbed shear conditions may shift the endothelium toward a proinflammatory and dysfunctional phenotype [2,3,4,5,6,7].

Cardiopulmonary bypass (CPB) profoundly alters the normal relationship between cardiac contraction and vascular blood flow. During beating-heart partial CPB, native cardiac ejection is retained and contributes to systemic pulsatility. In contrast, total CPB with aortic cross-clamping and cardioplegic arrest largely eliminates native cardiac pulsation, leaving systemic perfusion primarily dependent on pump-generated flow. CPB also introduces hemodilution, blood contact with artificial surfaces, nonphysiological shear forces, and ischemia–reperfusion-related inflammatory stimuli [8,9]. These changes may activate the vascular endothelium and contribute to postoperative inflammation and organ dysfunction. Nevertheless, the early endothelial-associated biomarker response to different CPB configurations remains incompletely characterized, particularly in veterinary-relevant large-animal models.

Vascular cell adhesion molecule-1 (VCAM-1) is an immunoglobulin-superfamily adhesion molecule that is upregulated on activated endothelial cells and promotes leukocyte adhesion and transendothelial migration through interaction with α4β1 integrin [10,11]. Endothelial VCAM-1 expression increases under low- or disturbed shear conditions, and its extracellular domain can be released into the circulation through proteolytic shedding, in part by a disintegrin and metalloproteinase-17 (ADAM-17) [3,11]. Circulating VCAM-1 has therefore been used as a marker of endothelial activation during cardiac surgery and may be more readily detectable than some other soluble adhesion molecules during acute vascular stress [12]. Simultaneous assessment of VCAM-1 and ADAM-17 may provide complementary information regarding endothelial activation and shedding-related processes.

Thrombospondin-1 (TSP-1) and platelet endothelial cell adhesion molecule-1 (PECAM-1) were included as additional exploratory markers. TSP-1 is a matricellular glycoprotein released by activated platelets and endothelial cells, and its expression can be influenced by disturbed flow and shear-related signaling [13,14,15,16,17,18,19]. PECAM-1 is expressed on endothelial cells, platelets, and leukocytes and forms part of the endothelial mechanosensory complex that transduces changes in wall shear stress [20,21,22,23,24]. Because these molecules participate in distinct but overlapping mechanobiological and inflammatory pathways, their combined evaluation may help determine whether CPB configuration is associated with systemic soluble-marker changes, tissue-level endothelial responses, or both.

We hypothesized that total CPB and partial CPB would be associated with different endothelial-associated biomarker responses. Accordingly, this exploratory comparative study measured serum VCAM-1, ADAM-17, and TSP-1 concentrations at serial peri-CPB time points and evaluated VCAM-1 and PECAM-1 immunoreactivity in vascular and renal tissues from juvenile pigs. The primary objective was to compare circulating VCAM-1 concentrations between groups at the end of the 60 min CPB period. Because the experimental configurations differed not only in terms of native pulsatility but also in aortic cross-clamping, cardioplegia, myocardial ischemia–reperfusion, and native cardiac output, the study was designed to evaluate the overall effect of CPB configuration rather than to isolate pulsatility as a single causal factor.

## 2. Materials and Methods

### 2.1. Experimental Design

Sixteen 8-week-old intact female piglets (three-way crossbred Landrace × Large White × Duroc) were randomly assigned by coin toss before the experiment to one of two groups: a partial CPB group (*n* = 8), in which CPB was performed without aortic cross-clamping while the heart continued beating, and a total CPB group (*n* = 8), in which aortic cross-clamping was followed by cardioplegic arrest induced using St. Thomas II solution (MIOTECTER Cardioplegic solution; Fuso Pharmaceutical Industries, Ltd., Osaka, Japan). Six animals (three per group) were subsequently euthanized: four because of persistent mean arterial pressure ≤40 mmHg and two because of deterioration in ventilation during tracheal intubation. Thus, five animals per group were included in the final analysis. The individual animal was considered the experimental unit.

Animals were sedated with medetomidine (40 μg/kg, intramuscularly), midazolam (0.2 mg/kg, intramuscularly), and butorphanol (0.2 mg/kg, intramuscularly). After placement of a 24-gauge intravenous catheter in an auricular vein, anesthesia was induced with propofol (5 mg/kg, intravenously). Animals were intubated with a 5-Fr endotracheal tube and mechanically ventilated at a respiratory rate of 12 breaths/min, a tidal volume of 10 mL/kg, and a positive end-expiratory pressure of 4 cm H_2_O. Anesthesia was maintained with a continuous propofol infusion (0.3 mg/kg/min), and rocuronium (0.6 mg/kg, intravenously) was administered for neuromuscular blockade. Invasive arterial blood pressure was monitored through a 22-gauge catheter placed in the internal carotid artery. Following initiation and stabilization of CPB, extracorporeal circulation was maintained for 60 min in both groups. In the total CPB group, aortic cross-clamping was performed and maintained for the 60 min CPB period after CPB had been established. After 60 min of CPB, the aortic cross-clamp was removed in the total CPB group, and animals in both groups were weaned from CPB. Protamine was administered 15–30 min later. Animals in both groups were euthanized within 30 min after discontinuation of CPB by intracardiac administration of potassium chloride (150 mg/kg). The study protocol was approved by the Rakuno Gakuen University Animal Experimentation Committee (protocol code VH24B2; approval date: 7 May 2025).

### 2.2. Cardiopulmonary Bypass Setup

Heparin (550 IU/kg, intravenously) was administered 3 min before initiation of CPB to maintain an activated clotting time of >400 s. The CPB circuit (MERA HP Exelung TPC, HPO-06RHF-CP; Senko Medical Instrument Mfg. Co., Ltd., Tokyo, Japan) was primed with 500 mL of lactated Ringer’s solution. Arterial cannulation was performed through the femoral artery using a 16-gauge hemodialysis needle (Seal Touch Cannula GA, 69-007; Nipro Corporation, Osaka, Japan), and venous drainage was established by cannulation of the right atrial appendage through a median sternotomy using a 22 Fr cannula. Venous return was gravity-assisted and augmented using a roller pump (HAS-P100; Senko Medical Instrument Mfg. Co., Ltd., Tokyo, Japan). Pump flow was maintained at ≥300 mL/min. Body temperature was maintained at 34 °C using an integrated heat exchanger (Low-Temperature Water Bath, LTB-250α, AS ONE Corporation, Osaka, Japan), and acid–base balance was managed using the α-stat strategy (i-STAT^®^Analyzer, Abbott Laboratories, Chicago, IL, USA).

### 2.3. Biomarker Measurements

**Figure 1:**
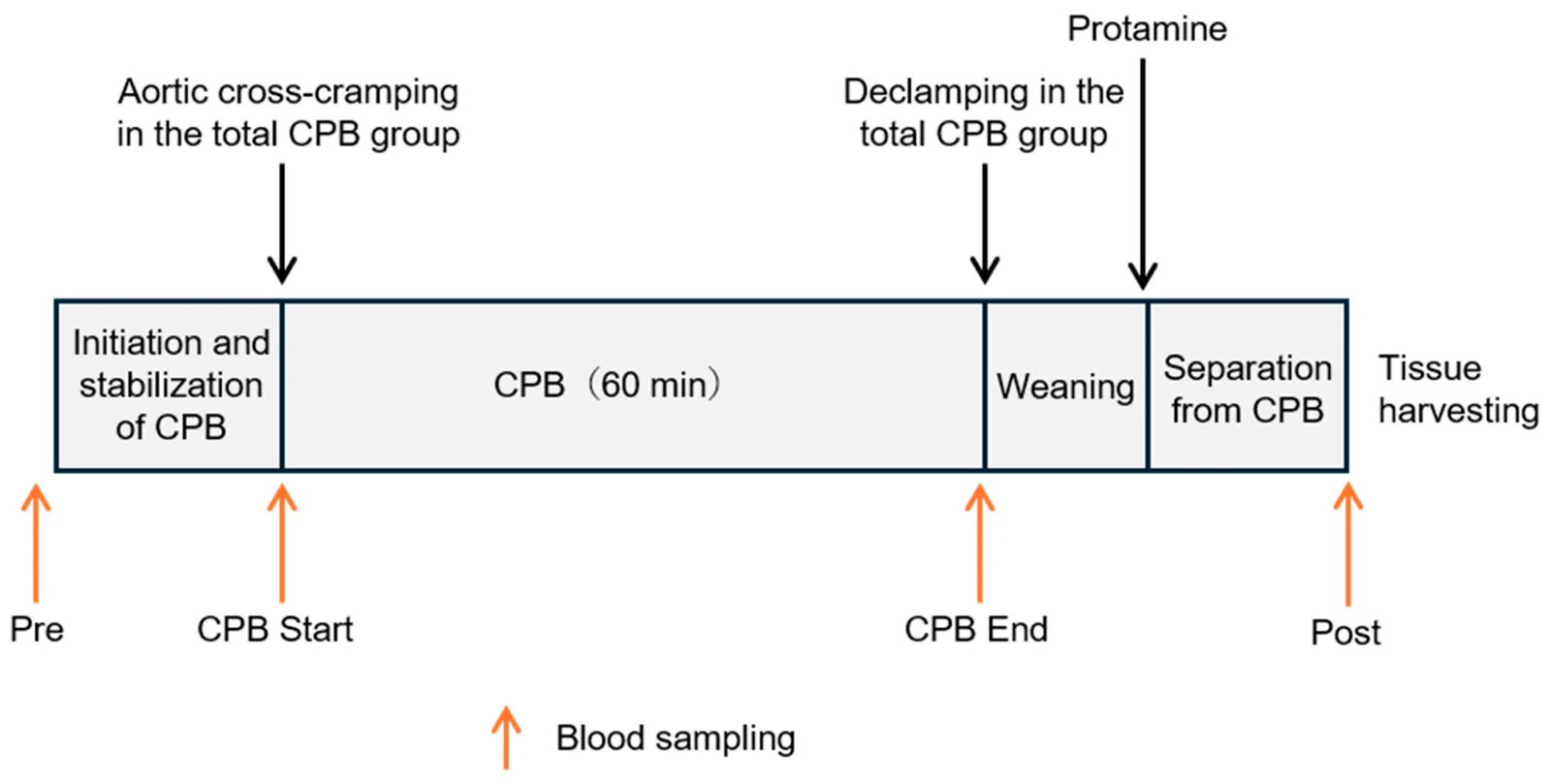
Experimental protocol. The figure illustrates the time points for serum and tissue sampling. CPB, cardiopulmonary bypass.

Blood samples were collected at four time points: before CPB (Pre), immediately after CPB initiation (CPB Start), at the end of the 60 min CPB period (CPB End), and after protamine administration (Post) (Figure 1). Serum was stored at −80 °C until analysis. Concentrations of VCAM-1 (Porcine VCAM-1 ELISA Kit, RDR-VCAM1-p; Reddot Biotech Inc., Houston, TX, USA), ADAM-17 (Pig ADAM-17 ELISA Kit, abx361925; Abbexa Ltd., Cambridge, UK), and TSP-1 (TSP-1 ELISA Kit, RE1923P; AS ONE Corporation, Osaka, Japan) were measured by enzyme-linked immunosorbent assay (ELISA) according to the manufacturers’ instructions. The reported detection ranges for the VCAM-1 and ADAM-17 assays were 15.625–1000 ng/mL and 62.5–4000 pg/mL, respectively, and the reported detection limit for the TSP-1 assay was 46.88 pg/mL. According to the manufacturers’ instructions, the intra-assay coefficients of variation were <10% for all assays. All samples for each biomarker were analyzed in the same assay run. Values outside the reported detection ranges were retained for analysis, as were duplicate measurements that did not meet the predefined quality control criteria. All samples were analyzed in duplicate, and the investigators performing the ELISA measurements were not blinded to group allocation.

### 2.4. Immunohistochemical Staining

After euthanasia, the aorta, pulmonary artery, caudal vena cava and left kidney were collected. Tissues were fixed in 4% paraformaldehyde, dehydrated through graded alcohols, embedded in paraffin, and sectioned at a thickness of 5 μm. Immunohistochemical staining was performed using monoclonal antibody against VCAM-1 (MA516429; Thermo Fisher Scientific, Waltham, MA, USA) and a polyclonal antibody against PECAM-1 (0195R; Thermo Fisher Scientific, Waltham, MA, USA).

### 2.5. Vcam-1 Staining

Deparaffinized sections were cleared in xylene, rehydrated, and incubated with 3% hydrogen peroxide for 30 min to block endogenous peroxidase activity. Sections were permeabilized with 0.05% Triton X-100 and subjected to antigen retrieval with pepsin in pH 9.0 buffer at 37 °C for 30 min. Nonspecific binding was blocked using Block Ace (KAC Co., Ltd., Kyoto, Japan) at 37 °C for 30 min. Sections were incubated overnight at 4 °C with the primary anti-VCAM-1 antibody diluted 1:100. After washing, Histofine Simple Stain MAX-PO (MULTI) (Nichirei Biosciences Inc., Tokyo, Japan) was applied for 30 min at room temperature. Color was developed for 10 min, and sections were counterstained with hematoxylin for 30 s.

### 2.6. Pecam-1 Staining

Deparaffinized sections were cleared in xylene, rehydrated, and subjected to antigen retrieval by autoclaving in pH 9.0 buffer at 121 °C for 20 min. Endogenous peroxidase activity was blocked with 3% hydrogen peroxide for 30 min. Sections were permeabilized with 0.05% Triton X-100 and incubated overnight at 4 °C with the primary anti-PECAM-1 antibody diluted 1:200. After washing, Histofine Simple Stain MAX-PO (MULTI) was applied for 30 min at room temperature. Color was developed for 10 min, and sections were counterstained with hematoxylin for 20 s.

All sections were evaluated by light microscopy by a single observer blinded to group assignment. Normal mouse serum was used as a negative control for VCAM-1 staining, and normal rabbit serum was used as a negative control for PECAM-1 staining, at the same dilution as the corresponding primary antibodies. Immunohistochemical staining was evaluated qualitatively based on the presence or absence of immunoreactivity in the relevant cell types. Samples were classified as positive when immunoreactivity was observed in the target cells and as negative when no immunoreactivity was observed. No predefined quantitative threshold was used for classification.

### 2.7. Statistical Analysis

This study was an exploratory pilot study, and no formal sample-size calculation was performed. However, the sample size was determined with reference to previous porcine CPB studies [25,26,27].

A single primary endpoint was defined as the between-group difference in serum VCAM-1 concentration at CPB End, before examination of the outcome data. ADAM-17 and TSP-1 were considered complementary exploratory biomarkers.

Between-group comparisons were performed using the Mann–Whitney U test, and within-group comparisons were performed using the Wilcoxon signed-rank test. Hodges–Lehmann estimates of the location shift and their 95% confidence intervals were also calculated for both between-group and within-group comparisons. Effect sizes were expressed as rank-biserial correlations for between-group comparisons and paired rank-biserial correlations for within-group comparisons, with 95% confidence intervals calculated using 10,000 bootstrap resamples. For between-group comparisons, positive effect sizes and Hodges–Lehmann estimates indicate higher values in the total CPB group than in the partial CPB group. For within-group comparisons across measurement time points, positive effect sizes and Hodges–Lehmann estimates indicate higher values at the later time point than at the earlier time point. Effect sizes were interpreted as small (|r| ≥ 0.1), medium (|r| ≥ 0.3), or large (|r| ≥ 0.5) [28]. Between-group comparisons at Pre, CPB Start, and Post, all within-group comparisons, and all analyses of ADAM-17 and TSP-1 were considered exploratory. No adjustment for multiple comparisons was applied; therefore, these results were interpreted cautiously. Immunohistochemical findings were summarized descriptively because of the small sample size and sparse positive staining. All tests were two-sided, and *p* < 0.05 was considered statistically significant. Statistical analyses were performed without blinding to group allocation using EZR version 1.68.

## 3. Results

### 3.1. Baseline Characteristics

Data are presented as medians [interquartile range]. Body weight did not differ between the partial and total CPB groups (7.95 [7.05–8.10] kg vs. 8.00 [7.95–8.05] kg, respectively; *p* = 0.92). Although changes in pump flow were not continuously recorded, it was maintained at ≥300 mL/min. When adequate venous drainage could not be maintained at this flow rate, additional fluid was administered and pump flow was temporarily increased. The pump flow under these conditions is provided in Supplementary Table S1. Total infused volume, including the priming solution, intraoperative fluids, and cardioplegia, was also comparable between groups (650 [600–700] mL vs. 666 [580–830] mL, respectively; *p* = 0.92). Pre-CPB, hematocrit values were similar (30 [29–33] % vs. 31 [30–32] %, respectively; *p* = 0.83) and hemoglobin concentration was 10.2 [9.9–11.2] g/dL vs. 10.5 [10.2–10.9] g/dL, respectively; *p* = 0.83. During CPB, hematocrit values were 17% and 16% in one animal in the partial CPB and total CPB groups, and hemoglobin concentration was 5.8 g/dL and 5.4 g/dL in same animal, whereas values in all other animals were below the detection limit because of hemodilution.

### 3.2. Circulating Endothelial Biomarkers

**Figure 2:**
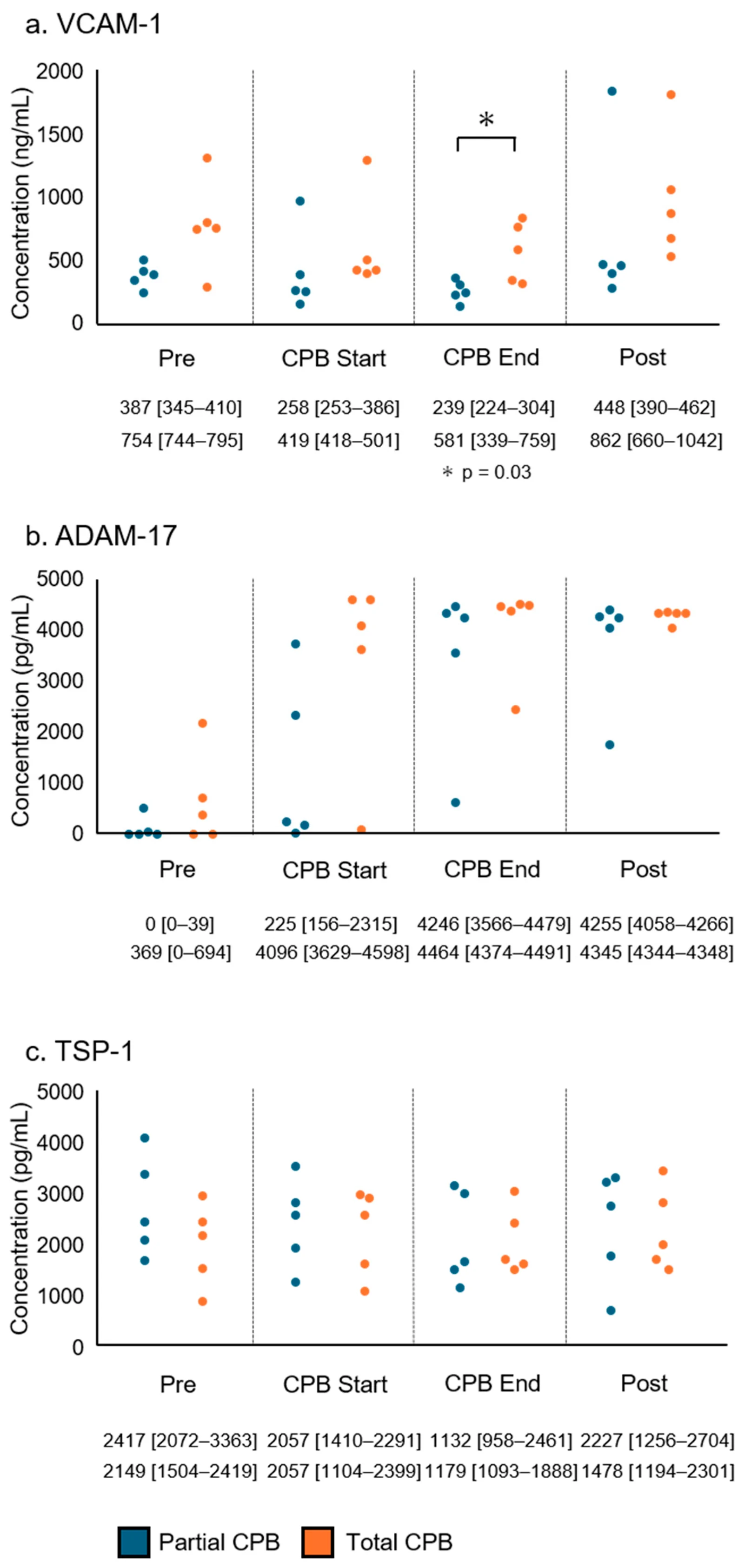
Comparison of serum concentrations of endothelial-associated biomarkers. Serum concentrations of (**a**) vascular cell adhesion molecule-1 (VCAM-1), (**b**) a disintegrin and metalloproteinase-17 (ADAM-17), and (**c**) thrombospondin-1 (TSP-1) measured at four time points: Pre, Cardiopulmonary bypass (CPB) Start, CPB End, and Post. A significant elevation in circulating VCAM-1 was observed at the end of CPB in the total CPB group compared with the partial CPB group (* *p* < 0.05). No significant intergroup differences were detected for ADAM-17 or TSP-1. Median [IQR] values for each biomarker are shown directly below each serum sampling time point. The upper row represents the partial CPB group, and the lower row represents the total CPB group.

The partial CPB group underwent CPB with continued cardiac beating and without aortic cross-clamping, whereas the total CPB group underwent aortic cross-clamping followed by cardioplegic arrest. At CPB End, serum VCAM-1 concentration was higher in the total CPB group than in the partial CPB group (581 [339–759] ng/mL vs. 239 [224–304] ng/mL; |rank-biserial r| = 0.84 (95%CI, 0.36 to 1.00); Hodges–Lehmann estimate of the location shift = 341.7 ng/mL (95% CI, 5.1–607.0 ng/mL); *p* = 0.03; Figure 2a). No significant between-group differences in VCAM-1 were detected at the other sampling time points. Serum ADAM-17 and TSP-1 concentrations showed no significant between-group or within-group differences (Figure 2b,c; Table 1 and Table 2). Serum concentrations of each biomarker are shown in Supplementary Table S2.

### 3.3. Immunohistochemistry

**Figure 3:**
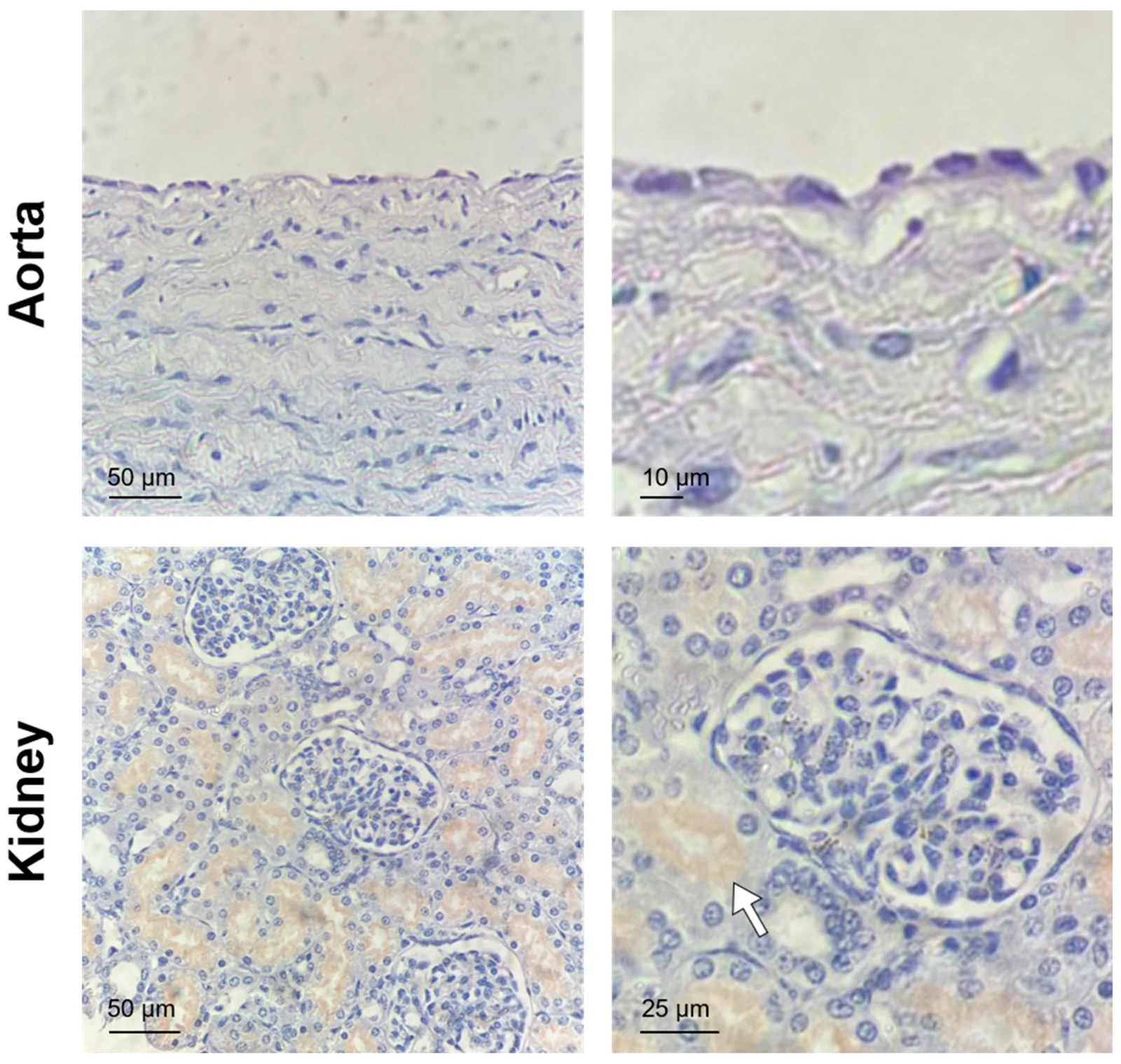
Tissue-specific vascular cell adhesion molecule-1 (VCAM-1) expression following cardiopulmonary bypass (CPB) exposure. Representative immunohistochemical staining for VCAM-1 in the aorta and kidney. Endothelial VCAM-1 expression was below the detection threshold in large vessels in both groups. In contrast, diffuse VCAM-1 positivity was observed in proximal tubular epithelial cells in one animal in the total CPB group (white arrow). Scale bars as indicated.

**Figure 4:**
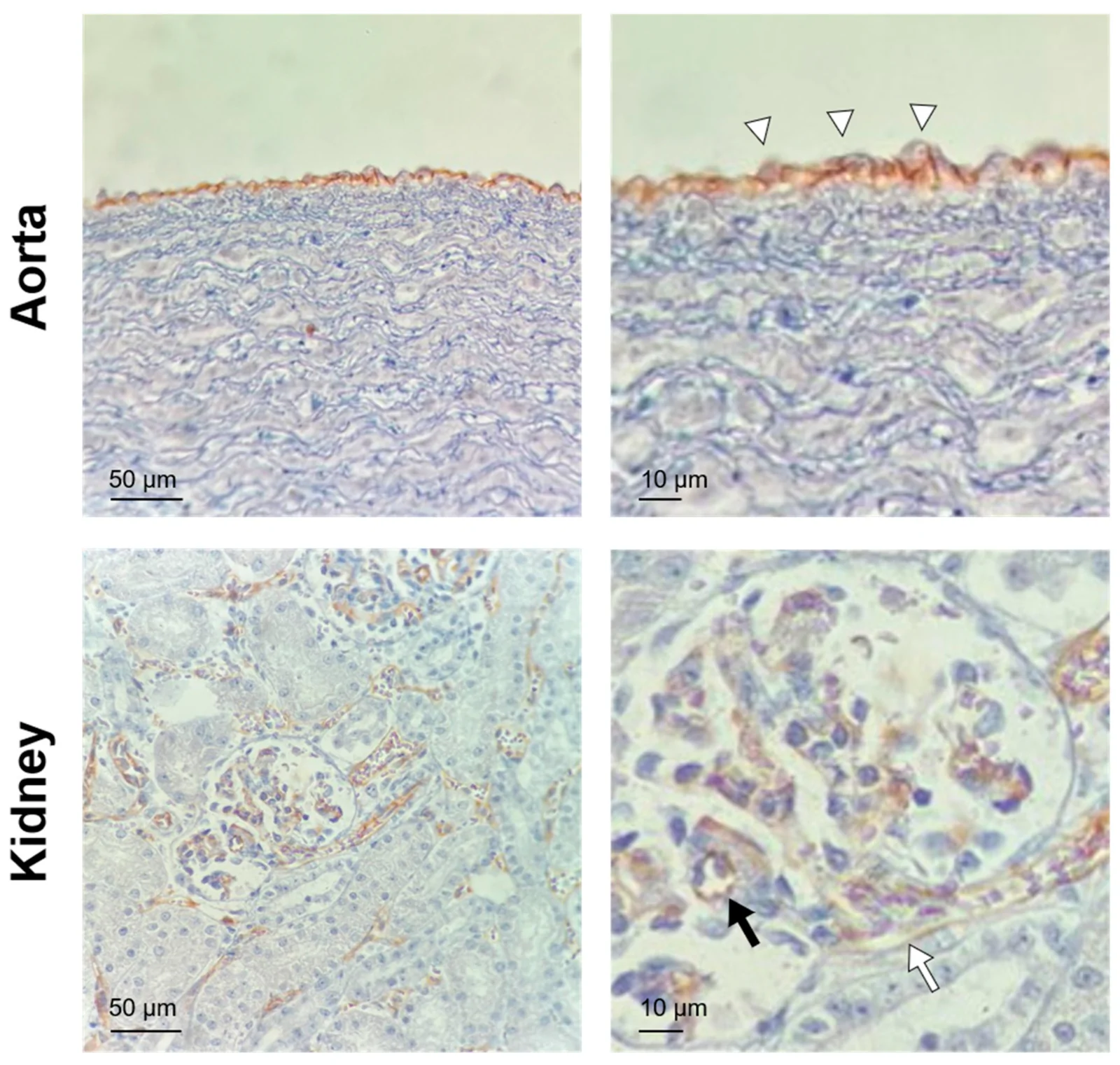
Platelet endothelial cell adhesion molecule-1 (PECAM-1) expression in vascular and renal tissues. Immunohistochemical staining for PECAM-1 demonstrating preserved endothelial expression in selected vascular beds in both cardiopulmonary bypass (CPB) groups, with no consistent intergroup difference. Representative images show endothelial positivity in the aorta (arrowhead), renal arterioles (white arrow), and glomerular capillaries (black arrow). Scale bars as indicated.

No VCAM-1 immunoreactivity was observed in vascular endothelial cells in either group. Diffuse VCAM-1 immunoreactivity was observed in proximal tubular epithelial cells in only one animal in the total CPB group (Figure 3; Table 3). This finding was considered an exploratory observation and was not interpreted as evidence of a group difference. PECAM-1 expression was detected in endothelial cells of selected vascular beds in two animals in each group (Figure 4; Table 4).

## 4. Discussion

This exploratory study compared early circulating endothelial-associated biomarker responses and tissue immunohistochemical findings in juvenile pigs undergoing partial CPB or total CPB.

The principal finding was a higher serum VCAM-1 concentration in the total CPB group at the end of the 60 min bypass period. In contrast, serum ADAM-17 and TSP-1 concentrations did not differ significantly between groups. No VCAM-1 immunoreactivity was observed in vascular endothelial cells in either group under the staining conditions used, and PECAM-1 staining showed no consistent intergroup difference. Taken together, these findings indicate that CPB configuration was associated with an early change in a soluble endothelial activation marker without a corresponding detectable difference in tissue expression during the observation period. However, given the small sample size and the wide confidence interval for the location-shift estimate, the magnitude of the effect remains imprecisely estimated. Thus, the VCAM-1 finding should not be interpreted based on statistical significance alone. In addition, tissue immunoreactivity was assessed qualitatively, and the sensitivity of the immunohistochemical staining procedure could not be fully validated in the present study; therefore, the absence of detectable tissue immunoreactivity should be interpreted with caution.

VCAM-1 is particularly relevant to this comparison because its endothelial expression is responsive to inflammatory stimuli and altered shear conditions [3,10,11,12]. In the partial CPB group, continued native cardiac contraction likely preserved a degree of pulsatile flow, whereas cardioplegic arrest in the total CPB group reduced native pulsatility. Additionally, animals in the total CPB group underwent aortic cross-clamping, cardioplegia, cardiac arrest, loss of native cardiac output, and subsequent myocardial ischemia–reperfusion, whereas those in the partial CPB group did not. Although the experimental and clinical literature suggest that flow pattern, in addition to mean flow or shear magnitude, can influence endothelial phenotype [2,3,4,5,6,7,9], each of these factors may influence systemic inflammation, endothelial activation, and soluble adhesion-molecule release. Consequently, the observed VCAM-1 difference should be interpreted as an association with the difference in CPB configuration between the partial and total CPB conditions, rather than as an effect attributable to differences in pulsatility alone.

Circulating VCAM-1 may increase because of enhanced endothelial expression, increased proteolytic shedding, reduced clearance, or a combination of these mechanisms. Although ADAM-17 contributes to VCAM-1 shedding [11], serum ADAM-17 did not differ between groups or correlate with VCAM-1. However, circulating ADAM-17 levels may not reflect local endothelial activity. Soluble VCAM-1 may reflect transient, spatially heterogenous, or low-level endothelial activation below the detection sensitivity of conventional immunohistochemistry. Differences in sampling time are also important: serum was obtained at CPB End, whereas tissues were collected after weaning from CPB and protamine administration, when endothelial expression may already have changed.

Hemodilution represents another potential influence on circulating biomarker concentrations during CPB. The priming volume was large relative to body size, and hematocrit values during bypass were frequently below the analytical detection range. At pre-CPB, hematocrit and total infused volume were comparable between groups; the inability to quantify hematocrit accurately during CPB prevented the adjustment of biomarker concentrations for plasma-volume change, and residual dilutional confounding cannot be excluded.

Diffuse VCAM-1 immunoreactivity was observed in proximal tubular epithelial cells in one animal in the total CPB group. Renal tubular epithelial cells respond to tubular fluid shear, hypoperfusion, inflammatory mediators, and ischemia–reperfusion-associated stress [29,30]. The finding is therefore biologically plausible in the setting of total CPB, but its occurrence in only one animal precludes broader interpretation. It should be regarded as an isolated exploratory observation.

No significant intergroup differences were detected for TSP-1. TSP-1 is influenced by platelet activation, disturbed flow, and tissue injury, but its response may depend on stimulus intensity and duration [13,14,15,16,17,18,19]. Similarly, PECAM-1 participates in endothelial mechanotransduction, but changes in phosphorylation, subcellular localization, or signaling activity may not be reflected by immunohistochemical staining [18,19,20,21,22]. Thus, the 60 min CPB period may have been insufficient to detect sustained changes. These negative findings should not be interpreted as evidence that the corresponding pathways were unaffected.

The clinical and translational significance of the VCAM-1 increase remains uncertain. CPB configuration has been discussed as a contributor to endothelial dysfunction, and clinical studies have investigated whether it improves postoperative recovery [9]. The present porcine data add preliminary evidence that total CPB may be accompanied by an early soluble endothelial-associated response. However, the study did not evaluate postoperative organ function, inflammatory cytokines, microvascular perfusion, or longer-term outcomes. It therefore remains unknown whether the observed biomarker difference represents a transient biochemical signal or a response with functional consequences.

This study has several limitations. First, the sample size was small, no formal a priori sample-size calculation was performed, and the estimates are consequently imprecise. The statistically significant VCAM-1 result arose from a single primary comparison but requires independent confirmation, whereas the absence of significant differences in ADAM-17 and TSP-1 cannot exclude biologically relevant effects. Second, pulsatility, wall shear stress, native cardiac output, and detailed hemodynamic waveforms were not measured directly. Third, the two experimental configurations differed simultaneously in several procedural factors, preventing causal attribution to pulsatility. Fourth, multiple exploratory comparisons were performed without multiplicity adjustment; these results should therefore be regarded as hypothesis-generating. Fifth, the pump flow was not continuously recorded, and although the changes were transient, the maximum pump flow varied between animals. Therefore, the possibility that these variations may have influenced the study outcomes cannot be excluded. Sixth, severe hemodilution limited the interpretation of intraprocedural hematocrit and prevented plasma-volume correction. Seventh, the relationships between study outcomes and arterial pressure, body temperature, heart rate, oxygenation status, the duration of cardioplegia, and lactate levels were not evaluated. Eighth, immunohistochemistry was based on a limited number of sampled tissues, a single post-CPB collection point, and qualitative observations with sparse positive findings. In addition, positive control tissues were not included. Ninth, a repeated-measures analysis incorporating group-by-time interaction was not performed. Finally, gene expression, endothelial microparticles, inflammatory cytokines, platelet activation markers, and organ-injury biomarkers were not assessed. Larger, prospectively powered studies incorporating direct hemodynamic measurements, serial tissue or molecular assessments, and postoperative functional outcomes are needed to validate and extend these findings.

## 5. Conclusions

In juvenile pigs, total CPB was associated with a higher circulating VCAM-1 concentration at the end of a 60 min CPB period than partial CPB, whereas ADAM-17, TSP-1, and tissue VCAM-1 and PECAM-1 findings showed no consistent intergroup differences. Because the CPB configurations differed in pulsatility and several other procedural and physiological factors, the VCAM-1 response cannot be attributed to reduced pulsatility alone. As this work was a pilot study, this preliminary finding should be interpreted cautiously and further mechanistic studies should be conducted using larger cohorts, considering direct assessment of flow pulsatility and wall shear stress, and with longer follow-up.

## Figures and Tables

**Table 1:** Comparison of serum biomarker concentrations at four time points between partial and total cardiopulmonary bypass (CPB) groups.

Biomarkers	Time Point	*p*-Values	Effect Size (95% CI)	Hodges–Lehmann Estimate (95% CI)
VCAM-1 (ng/mL)	Pre	0.10	0.68 (0.04 to 1.00)	384.9 (−94.8 to 912.3)
	CPB Start	0.10	0.68 (0.04 to 1.00)	163.8 (−537.3 to 1022.2)
	CPB End	0.03	0.84 (0.36 to 1.00)	341.7 (5.1 to 607.0)
	Post	0.15	0.60 (−0.20 to 1.00)	391.2 (−956.2 to 1340.1)
ADAM-17 (pg/mL)	Pre	0.37	0.60 (−0.04 to 1.00)	329.3 (−125.0 to 2177.9)
	CPB Start	0.15	0.60 (−0.20 to 1.00)	2294.3 (−167.9 to 4452.9)
	CPB End	0.22	0.52 (−0.28 to 1.00)	180.7 (−1806.4 to 3827.9)
	Post	0.42	0.36 (−0.44 to 1.00)	88.6 (−202.1 to 2590.7)
TSP-1 (pg/mL)	Pre	0.42	−0.36 (−0.92 to 0.44)	−785.6 (−2494.4 to 764.4)
	CPB Start	0.92	−0.04 (−0.76 to 0.76)	−187.5 (−1741.3 to 1320.0)
	CPB End	0.84	0.12 (−0.68 to 0.84)	46.3 (−1492.5 to 1375.6)
	Post	1.00	−0.04 (−0.84 to 0.68)	−61.9 (−1591.3 to 1658.1)

The *p*-values were calculated using the Mann–Whitney U test. Effect sizes were expressed as rank-biserial correlation coefficients, with 95% confidence intervals calculated using 10,000 bootstrap resamples. Hodges–Lehmann estimates of the location shift and their 95% confidence intervals were also calculated. Positive effect sizes and Hodges–Lehmann estimates indicate higher values in the total CPB group than in the partial CPB group. VCAM-1, vascular cell adhesion molecule-1; ADAM-17, a disintegrin and metalloproteinase-17; TSP-1, thrombospondin-1.

**Table 2:** Within-group comparisons of serum biomarker concentrations between different time points in the partial and total cardiopulmonary bypass (CPB) groups.

Biomarkers	Group	Time Point Comparisons	*p*-Values	Effect Size (95% CI)	Hodges–Lehmann Estimate (95% CI)
VCAM-1 (ng/mL)	Partial CPB	Pre vs. CPB Start	0.81	−0.20 (−1.00 to 0.73)	−23.3 (−357.9 to 569.7)
		CPB Start vs. CPB End	0.31	−0.60 (−1.00 to 0.20)	−121.5 (−652.5 to 95.8)
	Total CPB	Pre vs. CPB Start	0.19	−0.73 (−1.00 to 0.20)	−172.2 (−377.3 to 95.5)
		CPB Start vs. CPB End	1.00	−0.07 (−1.00 to 0.87)	−49.1 (−520.4 to 330.5)
		CPB End vs. Post	0.06	1.00 (1.00 to 1.00)	281.3 (210.6 to 1029.0)
ADAM-17 (pg/mL)	Partial CPB	Pre vs. CPB Start	0.10	1.00 (1.00 to 1.00)	1471.1 (156.4 to 3691.4)
		CPB Start vs. CPB End	0.06	1.00 (1.00 to 1.00)	2078.6 (635.7 to 4106.4)
	Total CPB	Pre vs. CPB Start	0.06	1.00 (1.00 to 1.00)	3024.3 (57.1 to 4240.7)
		CPB Start vs. CPB End	0.31	0.60 (−0.33 to 1.00)	368.9 (−107.2 to 2382.2)
		CPB End vs. Post	0.63	−0.33 (−1.00 to 0.60)	−138.6 (−320.7 to 1908.6)
TSP-1 (pg/mL)	Partial CPB	Pre vs. CPB Start	0.06	−1.00 (−1.00 to −1.00)	−661.9 (−1763.8 to −356.9)
		CPB Start vs. CPB End	0.63	−0.33 (−1.00 to 0.87)	−137.5 (−1874.4 to 403.8)
	Total CPB	Pre vs. CPB Start	0.13	−0.87 (−1.00 to −0.2)	−359.4 (−524.4 to 316.3)
		CPB Start vs. CPB End	0.81	−0.20 (−1.00 to 0.87)	−63.8 (−1430.6 to 628.8)
		CPB End vs. Post	0.63	0.33 (−0.87 to 1,00)	202.2 (−206.3 to 1026.3)

The *p*-values were calculated using the Wilcoxon signed-rank test. Effect sizes were calculated as paired rank-biserial correlation coefficients, with 95% confidence intervals calculated using 10,000 bootstrap resamples. Hodges–Lehmann estimates of the location shift and their 95% confidence intervals were also calculated. Positive effect sizes and Hodges–Lehmann estimates indicate higher values at the later time point than at the earlier time point. VCAM-1, vascular cell adhesion molecule-1; ADAM-17, a disintegrin and metalloproteinase-17; TSP-1, thrombospondin-1.

**Table 3:** Tissue-specific expression of vascular cell adhesion molecule-1 (VCAM-1) following partial and total cardiopulmonary bypass (CPB).

Cell Type	Tissue	Partial CPB (*n* = 5)	Total CPB (*n* = 5)
Endothelium	Aorta	0/5	0/5
	Pulmonary Artery	0/5	0/5
	Caudal Vena Cava	0/5	0/5
	Renal Arterioles	0/5	0/5
	Glomerular Capillaries	0/5	0/5
Epithelium	Proximal Tubule	0/5	1/5
	Distal Tubule	0/5	0/5
	Collecting Duct	0/5	0/5

Immunohistochemical detection of VCAM-1 in vascular endothelial and epithelial compartments across major vascular beds and renal structures. Results are expressed as the number of positive samples per group.

**Table 4:** Tissue-specific expression of platelet endothelial cell adhesion molecule-1 (PECAM-1) following partial and total cardiopulmonary bypass (CPB).

Cell Type	Tissue	Partial CPB (*n* = 5)	Total CPB (*n* = 5)
Endothelium	Aorta	2/5	2/5
	Pulmonary Artery	2/5	2/5
	Renal Arterioles	2/5	2/5
	Glomerular Capillaries	2/5	2/5
Epithelium	All Renal Segments	0/5	0/5

Immunohistochemical detection of PECAM-1 in vascular endothelial and epithelial compartments across major vascular beds and renal structures. Results are expressed as the number of positive samples per group.

## Data Availability

The original contributions presented in this study are included in the article/Supplementary Material. Further inquiries can be directed to the corresponding author.

## Supplementary Materials

The following supporting information can be downloaded at: https://www.mdpi.com/article/10.3390/vetsci13090861/s1, Table S1: Minimum and Maximum pump flow rates for each animal during cardiopulmonary bypass (CPB). Table S2: Serum concentrations of each biomarker measured in each group at each time point.

## Abbreviations

ADAM-17	A disintegrin and metalloproteinase-17
CPB	Cardiopulmonary bypass
ELISA	Enzyme-linked immunosorbent assay
PECAM-1	Platelet endothelial cell adhesion molecule-1
TSP-1	Thrombospondin-1
VCAM-1	Vascular cell adhesion molecule-1

## References

[1] Chatzizisis Y.S., Coskun A.U., Jonas M., Edelman E.R., Feldman C.L., Stone P.H. (2007). Role of endothelial shear stress in the natural history of coronary atherosclerosis and vascular remodeling: Molecular, cellular, and vascular behavior. J. Am. Coll. Cardiol..

[2] Chen L., Qu H., Liu B., Chen B.C., Yang Z., Shi D.Z., Zhang Y. (2024). Low or oscillatory shear stress and endothelial permeability in atherosclerosis. Front. Physiol..

[3] DeVerse J.S., Sandhu A.S., Mendoza N., Edwards C.M., Sun C., Simon S.I., Passerini A.G. (2013). Shear stress modulates VCAM-1 expression in response to TNF-α and dietary lipids via interferon regulatory factor-1 in cultured endothelium. Am. J. Physiol. Heart Circ. Physiol..

[4] Marchio P., Guerra-Ojeda S., Vila J.M., Aldasoro M., Victor V.M., Mauricio M.D. (2019). Targeting Early Atherosclerosis: A Focus on Oxidative Stress and Inflammation. Oxid. Med. Cell. Longev..

[5] Papaioannou T.G., Karatzis E.N., Vavuranakis M., Lekakis J.P., Stefanadis C. (2006). Assessment of vascular wall shear stress and implications for atherosclerotic disease. Int. J. Cardiol..

[6] Hou Y., Screen H.R.C., Knight M.M. (2025). Pulsatile low shear stress increases susceptibility to endothelial inflammation via upregulation of IFT and activation of YAP. APL Bioeng..

[7] Garcia-Polite F., Martorell J., Del Rey-Puech P., Melgar-Lesmes P., O’Brien C.C., Roquer J., Ois A., Principe A., Edelman E.R., Balcells M. (2017). Pulsatility and high shear stress deteriorate barrier phenotype in brain microvascular endothelium. J. Cereb. Blood Flow. Metab..

[8] Paparella D., Yau T.M., Young E. (2002). Cardiopulmonary bypass induced inflammation: Pathophysiology and treatment. An update. Eur. J. Cardiothorac. Surg..

[9] Yan W., Wang T., Wang J., Yang R., Zhang H., Zhang M., Ji B. (2025). Effects of pulsatile flow on postoperative recovery in adult cardiac surgery with cardiopulmonary bypass: A systematic review and meta-analysis of randomized controlled trials. Heliyon.

[10] Singh V., Kaur R., Kumari P., Pasricha C., Singh R. (2023). ICAM-1 and VCAM-1: Gatekeepers in various inflammatory and cardiovascular disorders. Clin. Chim. Acta.

[11] Troncoso M.F., Ortiz-Quintero J., Garrido-Moreno V., Sanhueza-Olivares F., Guerrero-Moncayo A., Chiong M., Castro P.F., Garcia L., Gabrielli L., Corbalan R. (2021). VCAM-1 as a predictor biomarker in cardiovascular disease. Biochim. Biophys. Acta (BBA)-Mol. Basis Dis..

[12] Eikemo H., Sellevold O.F., Videm V. (2004). Markers for endothelial activation during open heart surgery. Ann. Thorac. Surg..

[13] Gutierrez L.S., Gutierrez J. (2021). Thrombospondin 1 in Metabolic Diseases. Front. Endocrinol..

[14] Kaiser R., Frantz C., Bals R., Wilkens H. (2016). The role of circulating thrombospondin-1 in patients with precapillary pulmonary hypertension. Respir. Res..

[15] Holme P.A., Ørvim U., Hamers M.J., Solum N.O., Brosstad F.R., Barstad R.M., Sakariassen K.S. (1997). Shear-induced platelet activation and platelet microparticle formation at blood flow conditions as in arteries with a severe stenosis. Arterioscler. Thromb. Vasc. Biol..

[16] Nobili M., Sheriff J., Morbiducci U., Redaelli A., Bluestein D. (2008). Platelet activation due to hemodynamic shear stresses: Damage accumulation model and comparison to in vitro measurements. ASAIO J..

[17] Bongrazio M., Silva-Azevedo L.D., Bergmann E.C., Baum O., Hinz B., Pries A.R., Zakrzewicz A. (2006). Shear stress modulates the expression of thrombospondin-1 and CD36 in endothelial cells in vitro and during shear stress-induced angiogenesis in vivo. Int. J. Immunopathol. Pharmacol..

[18] Frangogiannis N.G., Ren G., Dewald O., Zymek P., Haudek S., Koerting A., Winkelmann K., Michael L.H., Lawler J., Entman M.L. (2005). Critical role of endogenous thrombospondin-1 in preventing expansion of healing myocardial infarcts. Circulation.

[19] Freyberg M.A., Kaiser D., Graf R., Buttenbender J., Friedl P. (2001). Proatherogenic flow conditions initiate endothelial apoptosis via thrombospondin-1 and the integrin-associated protein. Biochem. Biophys. Res. Commun..

[20] Chiu J.J., Chien S. (2011). Effects of disturbed flow on vascular endothelium: Pathophysiological basis and clinical perspectives. Physiol. Rev..

[21] Xia L., Zhang B., Sun Y., Chen B., Yu Z. (2021). Analysis of Syk/PECAM-1 signaling pathway in low shear stress induced atherosclerosis based on ultrasound imaging. Comput. Methods Programs Biomed..

[22] Xie X., Wang F., Zhu L., Yang H., Pan D., Liu Y., Qu X., Gu Y., Li X., Chen S. (2020). Low shear stress induces endothelial cell apoptosis and monocyte adhesion by upregulating PECAM-1 expression. Mol. Med. Rep..

[23] Falati S., Patil S., Gross P.L., Stapleton M., Merrill-Skoloff G., Barrett N.E., Pixton K.L., Weiler H., Cooley B., Newman D.K. (2006). Platelet PECAM-1 inhibits thrombus formation in vivo. Blood.

[24] Tzima E., Irani-Tehrani M., Kiosses W.B., Dejana E., Schultz D.A., Engelhardt B., Cao G., DeLisser H., Schwartz M.A. (2005). A mechanosensory complex that mediates the endothelial cell response to fluid shear stress. Nature.

[25] Fortier S., DeMaria R.G., Lamarche Y., Malo O., Denault A., Desjardins F., Carrier M., Perrault L.P. (2004). Inhaled prostacyclin reduces cardiopulmonary bypass-induced pulmonary endothelial dysfunction via increased cyclic adenosine monophosphate levels. J. Thorac. Cardiovasc. Surg..

[26] Qureshi S.H., Patel N.N., Murphy G.J. (2018). Vascular endothelial cell changes in postcardiac surgery acute kidney injury. Am. J. Physiol. Ren. Physiol..

[27] Stevens L.M., Fortier S., Aubin M.C., El-Hamamsy I., Maltais S., Carrier M., Perrault L.P. (2006). Effect of tetrahydrobiopterin on selective endothelial dysfunction of epicardial porcine coronary arteries induced by cardiopulmonary bypass. Eur. J. Cardiothorac. Surg..

[28] Cohen J. (1988). Statistical Power Analysis for the Behavioral Sciences.

[29] Frohlich E.M., Zhang X., Charest J.L. (2012). The use of controlled surface topography and flow-induced shear stress to influence renal epithelial cell function. Integr. Biol..

[30] Duan Y., Gotoh N., Yan Q., Du Z., Weinstein A.M., Wang T., Weinbaum S. (2008). Shear-induced reorganization of renal proximal tubule cell actin cytoskeleton and apical junctional complexes. Proc. Natl. Acad. Sci. USA.

