# Supplementary material for: Circulating Endothelial-Associated Biomarker Responses During Beating-Heart Partial Versus Cardioplegic Total Cardiopulmonary Bypass in Juvenile Pigs: An Exploratory Comparative Study
Source: Vet Sci. 2026 Aug 25;13(9):861. doi: 10.3390/vetsci13090861 (PMC13611692; doi:10.3390/vetsci13090861)
Supplement: Supplementary file 1 [file vetsci-13-00861-s001.zip › supplementary table S1.pdf]

Table S1. Minimum and maximum pump flow rates for each animal during cardiopulmonary bypass (CPB).

Group	No.	Pump Flow (ml/min)	
		Minimum	Maximum
Partial CPB	1	300	340
	2		300
	3		300
	4		300
	5		450
Total CPB	1	300	300
	2		400
	3		400
	4		550
	5		600
