# Supplementary material for: Circulating Endothelial-Associated Biomarker Responses During Beating-Heart Partial Versus Cardioplegic Total Cardiopulmonary Bypass in Juvenile Pigs: An Exploratory Comparative Study
Source: Vet Sci. 2026 Aug 25;13(9):861. doi: 10.3390/vetsci13090861 (PMC13611692; doi:10.3390/vetsci13090861)
Supplement: Supplementary file 1 [file vetsci-13-00861-s001.zip › supplementary table S2.pdf]

Table S2. Serum concentrations of each biomarker measured in each group at each time point

		Pre		CPB Start		CPB End		Post	
Biomarkers	No.	Partial CPB	Total CPB	Partial CPB	Total CPB	Partial CPB	Total CPB	Partial CPB	Total CPB
VCAM-1 (ng/mL)	1	248.9	292.2	257.8	387.7	353.6	338.6	1818.7	659.9
	2	505.8	744.4	147.9	419.4	224.3	581.1	462.1	862.5
	3	344.8	1299.4	253.5	1279.9	132.0	759.5	390.3	1788.5
	4	409.7	794.6	386.4	417.3	239.5	309.3	448.4	521.3
	5	387.0	754.4	956.7	500.8	304.2	831.3	268.8	1041.9
ADAM-17 (pg/mL)	1	0.0	0.0	225.0	57.1	4331.4	2439.3	4265.7	4347.9
	2	0.0	0.0	0.0	3628.6	635.7	4463.6	1754.3	4353.6
	3	39.3	693.6	3730.7	4095.7	4478.6	4373.6	4396.4	4052.9
	4	0.0	368.6	156.4	4609.3	3565.7	4512.1	4057.9	4345.0
	5	493.6	2177.9	2315.0	4597.9	4245.7	4490.7	4255.0	4343.6
TSP-1 (pg/mL)	1	4054.8	2418.5	2291	2057.3	2624.8	2507.3	2704.1	2301
	2	2416.6	2149.1	2057.2	2465.4	2461	1887.9	2785.4	2914.1
	3	1654.1	868.5	737.2	549.8	599.8	1178.5	199.8	1194.1
	4	2071.6	2923.5	1409.8	2399.1	957.9	968.5	1256	1477.9
	5	3362.9	1504.1	3006	1104.1	1131.6	1092.3	2226.6	987.3

Serum concentrations were measured by enzyme-linked immunosorbent assay (ELISA). CPB, cardiopulmonary bypass; VCAM-1, vascular cell adhesion molecule-1; ADAM-17, a disintegrin and metalloproteinase-17; TSP-1, thrombospondin-1.
